# Single nucleotide polymorphisms (SNPs) are inherited from parents and they measure heritable events

**DOI:** 10.1186/1477-3163-4-2

**Published:** 2005-01-17

**Authors:** Kari Hemminki, Asta Försti, Justo Lorenzo Bermejo

**Affiliations:** 1 Division of Molecular Genetic Epidemiology, German Cancer Research Center (DKFZ), Im Neuenheimer Feld 580, D-69120 Heidelberg, Germany; 2 Department of Biosciences at Novum, Karolinska Institute, 141 57 Huddinge, Sweden

**Keywords:** genotyping, interactions, genomics, multiple comparisons, molecular epidemiology.

## Abstract

Single nucleotide polymorphisms (SNPs) are extensively used in case-control studies of practically all cancer types. They are used for the identification of inherited cancer susceptibility genes and those that may interact with environmental factors. However, being genetic markers, they are applicable only on heritable conditions, which is often a neglected fact. Based on the data in the nationwide Swedish Family-Cancer Database, we review familial risks for all main cancers and discuss the evidence for a heritable component in cancer. The available evidence is not conclusive but it is consistent in pointing to a minor heritable etiology in cancer, which will hamper the success of SNP-based association studies. Empirical familial risks should be used as guidance for the planning of SNP studies. We provide calculations for the assessment of familial risks for assumed allele frequencies and gene effects (odds ratios) for different modes of inheritance. Based on these data, we discuss the gene effects that could account for the unexplained proportion of familial breast and lung cancer. As a conclusion, we are concerned about the indiscriminate use of a genetic tool to cancers, which are mainly environmental in origin. We consider the likelihood of a successful application of SNPs in gene-environment studies small, unless established environmental risk factors are tested on proven candidate genes.

## Introduction

Genetic association studies on complex diseases have become very popular and most of them are case-control studies using single nucleotide polymorphisms (SNPs) as markers. There has been concern about the poor reproducibility of the results and the reasons for such discrepancies have been discussed [1-5]. However, the theoretical underpinnings of such studies have attracted less attention, apart from the use of SNPs as mapping tools, an application which we will not discuss in the present article [6-8]. Heritable etiology in many common diseases may not be overwhelming, and the use of genetic tools to dissect disease causation may thus be questionable. For cancer, all useful etiological measures, such as incidence changes upon time and migration, and aggregation of cancer among twins and families, point to a predominant environmental contribution to cancer causation [9-15]. However, in these contexts, the environment is anything that is not inherited, including variables that can be measured in epidemiological studies, in addition to un-measurable and random, stochastic events. The fact that only a minority of smokers are diagnosed with lung cancer is often cited as evidence for inherited differences in susceptibility to tobacco carcinogenesis. There are also other possible reasons, such as time-dependent stochastic effects, which in inbred animals are the likely reason that only some animals develop cancer when exposed to a constant level of a carcinogen.

How feasible is it then to carry out SNP studies on cancer, particularly when the subjects are overwhelmingly unselected cases, among whom familial cases are rare. It is worrisome that genotyping is now almost a required standard component in epidemiological studies, without consideration of the expected heritable influence. Formal sample size and power calculations are irrelevant if there are no data on the assumed heritable component in the causation of a particular cancer. It is also worrisome that the fundamental differences between purely genetic and gene-environment studies regarding control populations and multiple testing problems are not appreciated. In this contribution, we first review study designs and aims of SNP studies, then we give data on familial risks for the main types of cancer, to give an idea of the upper bounds for the risks that can be expected in genotyping studies. Finally, we will calculate familial risks resulting from variants of assumed genotype relative risk and allele frequency. Such data are useful in the assessment of study designs and in the evaluation of obtained results. We use the term 'familial' to denote cancers in two or more first-degree relatives and 'heritable' when an inherited gene defect is known or inferred due to a high risk [16]. Instead of 'genotype relative risk', we refer to 'odds ratio' (OR), consistent with terminology of most association studies.

## Study designs and aims

In the simplest form, a SNP with a known or assumed function is selected, and the genotypes are determined in cases and controls to test for association. Most studies published on cancer are of this kind, purely "genetic studies" testing the effect of the genotypes on the risk of cancer, without considering any other variables [17,18]. In addition to genotype effects, some studies have incorporated the effects of haplotypes and data on functional effects of the studied SNPs [19-21]. Population stratification has been an issue in association studies and it is important that the control subjects are drawn from the same ethnic and geographic population [2,5]. However, there is no need for individually based matching (age, gender etc.), typical of epidemiological case-control studies, as long as the genotypes of the control population follow the Hardy-Weinberg equilibrium [22,23]. Multiple testing is an issue in "genetic studies" but solutions are available, for example using the Bonferroni adjustment [2,5].

Most studies have selected SNPs with assumed functional effects or they test the effects of haplotypes. For some genes, null or truncating alleles exist and homozygotes would then lack a functional protein. However, for missense types of SNPs the functional effects may be small or nil when tested in *in vivo *systems [21]. Unfortunately, for many genes an *in vivo *functional test cannot be easily devised. Drug metabolism genes are a fortunate exception in this regard, because aberrant responses in humans have lead to the characterization of the underlying gene variants. One common feature of almost all the published studies is that patients have been collected without regard of a family history, thus sacrificing statistical power but attempting to compensate with a large sample size [24,25]. Using familial cases would be advantageous statistically, and some effects, such as that of CHEK2*1100delC, have only been detected among familial cases [26].

A variant of the candidate gene approach is the "gene-environment study", which is founded on the assumption that in complex diseases environmental factors interact with heritable factors, and a strong effect can be detected when both are present [27-29]. In epidemiology, interactions (also called effect modifications) are best described for multiple exposures, which may be additive, multiplicative or mixed [30]; these probably also apply to gene-environment interactions, but there are few bonefied examples on quantified gene-environment interactions [29]. It is conceptually appealing to assume that environmental factors interact with the genetic make-up to cause a differential susceptibility to cancer. However, examples are needed in order to verify this concept and its magnitude in cancer causation. The SNP component has become a favored adjunct to epidemiological studies, promoted with the hypothesis that the small, perhaps insignificant effects noted between exposure and cancer can be salvaged by incorporation genetic host factors into the study. These studies always include multiple comparisons, firstly, including the epidemiological variables in various classes, which may add up to thousands of cells (a small study with 5 variables in 5 strata each results in 5^5 ^= 3125 unique cells), and, secondly, the genes that are selected for analysis, are taken from a pool of tens or hundreds of potential candidate genes. No solutions have been found for this "two-dimensional" multiple testing problem. However, important for the present discussion, gene-environment interactions only exist if there is a heritable component in the particular cancer, and the likelihood of observing an effect is larger if the heritable component is large.

In populations of random mating, it may be plausible that gene-environment interactions of epidemiologically measurable magnitude exit in the absence of a measurable familial risk. In the case of many exposures, some with harmful and others with protective effects, interacting with many genes, it may be possible that the familial risks are missed in spite of true gene-environment effects. Similarly, non-conventional dose-effect relationships, such as those suggested for blood vitamin D levels and prostate cancer [31], would be difficult to reconcile in terms of any genetic models.

According to the complex disease paradigm, many relatively common alleles, interacting with environmental factors, cause susceptibility to common diseases [28,32]. Such a "non-Mendelian" inheritance may not cause an appreciable familial risk because the penetrance is so low that the likelihood of several family members being affected would be small. Twin studies should be able to assess the contribution of polygenic heritability [33], and the heritability estimates derived for colorectal (35% heritability), breast (27%) and prostate (42%) cancers, the only significant ones among site-specific cancers, encompass the total (broad) heritability, as definable using the twin model [11]. The much smaller heritability estimates, generated from family studies, were thought to result in part because of inability to consider such polygenic effects [13].

## Familial risks and proportions

Familial risk of a disease is a measure of its clustering in family members. Commonly, familial risk is defined between those who have a relative (e.g., parent or sibling) with cancer compared to those whose relatives are free from cancer, given as a familial relative risk or familial standardized incidence ratio (SIR). The SIRs shown below have been obtained from the Swedish Family-Cancer Database, the largest dataset of the kind in the world [34]. Familial SIRs have been adjusted for age, socio-economic status, period and region, and for women, for reproductive parameters. Table 1 shows familial SIRs for 0 to 68 year old offspring whose parents had the same cancer [35]. Table 1 also shows the number of observed cases, 95% confidence intervals (95%CIs) for the SIRs and the familial proportions, i.e., the percentage of all affected offspring who have an affected parent. A total of 4938 concordant familial cancers were found, with an overall SIR of 2.02. All the site-specific familial risks were significantly increased, except those for connective tissue. Hodgkin's disease showed the highest SIRs of 4.91, followed by testicular (4.35) and non-medullary thyroid cancers (3.26). Esophageal and ovarian cancers and multiple myeloma had SIRs in excess of 3.00. Among common cancers, the SIRs were increased for female breast (1.84), prostate (2.45) and colorectal adenocarcinoma (1.86), and the number of familial pairs ranged between 681 and 1779 for these common cancers. Apart from colorectal, breast and ovarian cancer, hardly anything is known about the genetic bases of these neoplasms, which should be good news to those who want to test the effects of candidate genes [36,37].

**Table 1:** SIR for cancer in offspring by parental concordant cancer

Cancer site	O	SIR	95%CI		Familial proportion, %
Breast	1779	**1.84**	1.76	1.93	8.5
Prostate	922	**2.45**	2.29	2.61	15.4
Colorectum (adenocarcinoma)	681	**1.86**	1.73	2.01	10.1
Lung	365	**2.09**	1.88	2.32	6.6
Melanoma	166	**2.62**	2.23	3.05	2.5
Urinary bladder	117	**1.75**	1.45	2.10	3.9
Nervous system	112	**1.75**	1.44	2.11	1.8
Ovary	97	**3.15**	2.56	3.85	3.3
Endometrium	83	**2.47**	1.97	3.07	2.9
Stomach	82	**2.17**	1.72	2.69	5.5
Skin, squamous cell	77	**2.52**	1.99	3.15	4.1
Non-Hodgkin's lymphoma	74	**1.82**	1.43	2.29	2.1
Kidney	64	**1.87**	1.44	2.39	2.8
Leukemia	55	**1.88**	1.42	2.45	1.7
Pancreas	46	**1.87**	1.37	2.49	3.0
Upper aerodigestive tract	39	**1.71**	1.22	2.35	1.9
Cervix	39	**1.82**	1.29	2.49	1.7
Endocrine glands	38	**2.22**	1.57	3.06	1.5
Liver	37	**1.66**	1.17	2.28	2.6
Myeloma	23	**3.32**	2.10	5.00	2.6
Thyroid gland, nonmedullary	12	**3.26**	1.68	5.71	1.0
Testis	10	**4.35**	2.07	8.04	0.5
Esophagus	8	**3.14**	1.34	6.22	1.3
Hodgkin's disease	8	**4.91**	2.10	9.73	0.7
Connective tissue	4	1.87	0.49	4.84	0.5
All	4938	**2.02**	1.97	2.08	5.5

Bold type: 95%CI does not include 1.00.

Familial proportion: % of affected offspring with affected parent

Familial cases constituted 15.4% of all prostate cancers, which was the largest proportion by far. The proportion was 10.1% for colorectal adenocarcinoma and 8.5% for breast cancer, but only 0.5% for connective tissue and testicular tumors. The proportions are generally higher for common cancers. Overall familial cancers (same cancer in offspring and parents) constituted 5.5% of all cancers.

We have tried to estimate the degree of environmental contribution to the familial risk by comparing cancer risks betweens spouses. Spouse concordance, which does not generally exceed an SIR of 1.3, can be noted only for cancers with known strong environmental risk factors: lung and genital cancers and early onset gastric and pancreatic cancers and melanoma [38,39]. Spouse correlation does not consider environmental sharing early in the life; this has been estimated by comparing cancer risks between siblings with a small or large age difference, respectively [40]. For most sites, including the breast and the colorectum, heritability is likely to be the main contributor to familial cancer [41,42]. Environmental factors are probably a large contributor to the familial aggregation of cervical, lung and upper aerodigestive tract cancers, and a minor contributor to familial risks for melanoma and squamous cell skin cancer [43,44].

If environmental causes of familial clustering have been quantified or excluded, familial SIRs and proportions give estimates on the heritable effects for cancer at the level of nuclear families (here between parents and offspring). Because of low penetrance, familial proportions underestimate true heritable effects. On the other hand, the twin model assumes that the shared environmental effects of monozygotic and dizygotic twins are identical, which may not be true. If monozygotic twins share more than dizygotic twins, the estimated heritability is exaggerated. Thus, the heritability estimates for cancer are still unreliable, and, due to possible interactions, a dichotomous classification into heritable and environmental components is conceptually inaccurate [45]. Moreover, the current models for twin studies do not allow the existence of interactions, a condition probably violated for many cancers. Nevertheless, the available data suggest that the heritability is low for most cancers, and even for prostate, breast and colorectal cancer it contributes a small etiological proportion.

## Familial risks from snps

Results from a successful SNP study can imply that the particular variant contributes to a familial risk of the particular cancer. The resulting familial risk depends on the allele frequency of the SNP, observed OR and the mode of inheritance, i.e., on the relative risks of heterozygotes compared to homozygotes. In the dominant model, the risk of heterozygotes equals that of the variant homozygotes; in the recessive model, the risk of heterozygotes equals that of the wild type homozygotes. In the additive model, the risk of heterozygotes is the mean of the two homozygotes; in the multiplicative model, the risks between the genotypes differ by a constant multiplier.

The methods for the calculation of familial risks to offspring of affected parents (comparable to SIRs of Table 1), based on allele frequency and OR of the genotype are presented elsewhere [46]. According to Table 2, the calculated familial risk is negligible at very low and very high allele frequencies when ORs are below 10, and at any allele frequency when OR is 2 or less. Most SNP studies are carried out on variants with frequencies at 5% or higher, and then substantial familial risks may be caused by a single gene with a high OR. Familial risk of breast cancer was 1.84 in Table 1; however, because the known genes, including *BRCA1/2*, *ATM*, *p53 and CHEK2*, explain about 25% of the risk [47], the unexplained familial risk is about 1.6. In Table 2 we have fold-faced SIRs that are incompatible with the empirical data for breast cancer (risk 1.60 or more), i.e., the resulting familial SIRs would be too high. If a single dominant gene would explain all the remaining familial risk of breast cancer, the allele frequency should be 0.2 and OR about 15; with allele frequency of 0.01, OR should be about 10. In the more likely scenario, many genes contribute to the familial risk, but their joint effect cannot exceed the above values.

**Table 2:** Familial risk to offspring of affected parents assuming define allele frequencies and their effects according various genetic models

	OR				
Allele frequency	1.5	2	5	10	20
Dominant model					
0.001	1.00	1.00	1.02	1.08	1.33
0.01	1.00	1.01	1.13	1.57	**2.84**
0.05	1.01	1.04	1.36	**1.99**	**2.90**
0.1	1.02	1.05	1.38	**1.80**	**2.24**
0.2	1.02	1.06	1.28	1.46	**1.60**
0.3	1.02	1.05	1.18	1.27	1.33
0.4	1.01	1.03	1.11	1.15	1.18
0.5	1.01	1.02	1.06	1.08	1.10
					
Additive model					
0.001	1.00	1.00	1.00	1.02	1.09
0.01	1.00	1.00	1.04	1.17	**1.63**
0.05	1.00	1.01	1.13	1.46	**2.13**
0.1	1.01	1.02	1.18	1.50	**1.97**
0.2	1.01	1.03	1.20	1.41	**1.63**
0.3	1.01	1.03	1.17	1.31	1.42
0.4	1.01	1.03	1.14	1.23	1.29
0.5	1.01	1.03	1.11	1.17	1.20
					
Recessive model					
0.001	1.00	1.00	1.00	1.00	1.00
0.01	1.00	1.00	1.00	1.00	1.00
0.05	1.00	1.00	1.00	1.01	1.04
0.1	1.00	1.00	1.01	1.06	1.23
0.2	1.00	1.01	1.08	1.28	**1.75**
0.3	1.00	1.02	1.16	1.47	**1.93**
0.4	1.01	1.03	1.23	1.52	**1.85**
0.5	1.01	1.04	1.25	1.48	**1.68**
					
Multiplicative model					
0.001	1.00	1.00	1.00	1.00	1.01
0.01	1.00	1.00	1.01	1.04	1.11
0.05	1.00	1.01	1.06	1.18	1.42
0.1	1.00	1.01	1.11	1.28	**1.60**
0.2	1.01	1.02	1.16	1.36	**1.67**
0.3	1.01	1.03	1.17	1.36	**1.61**
0.4	1.01	1.03	1.16	1.32	1.51
0.5	1.01	1.03	1.15	1.27	1.40

Because the prevalence has no effect on the calculated familial risks, Table 2 can be used for any cancers of variable prevalences. The familial SIR for lung cancer was 2.09 (Table 1). However, judged from the spouse correlation, probably a large but undefined proportion of familial risk for lung cancer can be explained by environmental factors, and the unexplained heritable component may be not very different from breast cancer. For upper aerodigestive tract cancers, the familial SIR was 1.71, but tobacco smoking and other environmental factors probably contribute to familial clustering and the heritable component is likely to be relatively small in this cancer.

It is of interest to examine the magnitude of familial risks which would be predicted from the published ORs for candidate genes. In a review of 34 polymorphisms in 18 different genes tested for breast cancer, a large proportion of the associations were not significant and the ORs were below 2.0 [17]. Even many significant ORs were below 2.0 and the resulting familial risk is negligible. However, there were some exceptions; in one study, *TNF-alpha *with an allele frequency of 0.2 showed an additive risk of about 10 (homozygote/homozygote). According to Table 2, the resulting familial risk would be about 1.4, i.e., if the effect were true, this gene would explain half of all familial risk for breast cancer; in that respect it would be two times more important than *BRCA1 *and *BRCA2 *combined. The effects of metabolic polymorphisms on various cancers have been reviewed in an IARC publication [48]. Among many genes, CYP2D6 has been analyzed in many studies as a risk factor for lung cancer, although it is not expressed in lung tissue [49,50]. Some genotyping studies have reported ORs between 5 and 15 for poor metabolizer genotypes. Assuming an allele frequency of 0.02 and a dominant OR of 10, Table 2 gives a familial risk of about 1.8, which, if true, would account for all familial risk of lung cancer not explainable by environmental factors.

## Conclusions

The poor reproducibility of candidate gene studies has most commonly been associated to small sample size, population stratification and low prior probability, i.e., poor selection of genes or SNPs; a SNP with small functional effect would also imply a low prior probability for an effect [5,32]. We agree that the low prior probability is an important factor but we would like to widen the scope of the query from the right gene to the right tool: is the genomic tool generally applicable to a disease that is mainly environmental? It is likely that some successes will continue to come in associating new genes with cancers of a reasonable heritable component, such as that of CHEK2*1100delC in breast cancer, and populations of familial cancers will be important either in finding the initial association or in confirming the effect. In spite of the unsolved multiple testing problems, we consider plausible that gene-environment interactions will be established between demonstrated risk factors and proven candidate genes, for which tobacco-induced lung cancer would appear an obvious choice; however, even the candidate genes for lung cancer are still being searched. Testing of unproven genes and/or unproven environmental factors for gene-environment interactions is the high-risk design for a multiple testing outcome. It is worrisome to the field of gene-environment interactions that no such proof-of-principle has yet been demonstrated.

It is commendable that all available molecular and environmental data are being used in attempts to understand the mechanisms of human cancer [51-53]. With increasing understanding of the cellular mechanisms more useful tools will become available. Even though these cellular systems are governed by heritable genes, variants in these genes may not have an impact large enough to predispose to heritable cancer. With current technological resources there is a growing danger that technology rather than biology is becoming the driving force in population studies [3]. Although the new technologies will allow benefits for the analysis of multiple SNPs and haplotypes in genetic pathways rather than in individual genes, they will not change the fact that cancer is mainly an environmental disease, expressed as somatic alterations on a heritable background. Forcing unproven genetic paradigms into all epidemiological studies is risky, and the likelihood of contradictory results may increase, leading to a gradual erosion of credibility.
